# Mutation spectrum of *CYP21A2* and correlation between genotype – phenotype in 81 Vietnamese patients with congenital adrenal hyperplasia due to 21-hydroxylase defficiency

**DOI:** 10.1186/1687-9856-2013-S1-P128

**Published:** 2013-10-03

**Authors:** Vu Chi Dung, Tran Van Khanh, Maki Fukami, Le Thi Phuong, Nguyen Thi Ha, Nguyen Thanh Liem, Ta Thanh Van

**Affiliations:** 1 Vietnam National Hospital of Pediatrics, Hanoi, Vietnam; 2 Hanoi Medical University, Hanoi, Vietnam; 3 National Research Institute for Child Health and Development, Tokyo, Japan

## 

Congenital adrenal hyperplasia (CAH) is one of the most common inherited metabolic disorders. It includes a group of autosomal recessive disorders caused by the deficiency of one of the enzymes involed in one of the various steps of adrenal steroid synthesis. The most common form of CAH (95%) is caused by mutations in *CYP21A2*, the gene encoding the adrenal steroid 21-hydroxylase enzyme (P450c21). Two major phenotype are recognized in 21-OHD: classic and non-classic. Classic CAH is clinically categorized in two groups: the simple-virilising and the salt-wasting form. The National Hospital of Pediatrics (NHP) in Hanoi is an 1100 bed tertiary referral centre servicing approximately 30 million people from northern provinces of Vietnam. At the start of 1999 there were 91 children with CAH due to 21-hydroxylase deficiency (21-OHD) managed at NHP. By June 2012 this increased to 624 [98.2% due to 21-OHD], representing a more than six fold increase over 12 years. Number of new cases ranged from 40 to 70 per year.

We aim to determine the mutations in the *CYP21A2* gene in Vietnamese patients with CAH and attempt a genotype-phenotype correlation. Molecular analysis was performed using PCR, multiplex ligation-dependent probe amplification and direct sequencing of PCR products of the *CYP21A2* gene in 81 CAH patients (39 male and 42 female). Correlation between phenotype and genotype was evaluated based on identified mutations and clinical manifestations.

Mutations were identified in 92.6% mutant alleles, 22 genotypes were found in 81 cases. Seven different causative mutations were identified in *CYP21A2* including one novel mutation. The most frequent genetic defect in the classic salt-wasting and simple virilizing forms was the IVS2-13A/C>G (54 alleles; 36%) mutation, followed by Large lesion (42 alleles; 28%) including exon 1 deletion (2 alleles), exon 1-3 deletion (10 alleles), exon 1-6 deletion (4 alleles), exon 1-8 deletion (2 alleles) and larger deletion (24 alleles); p.R356W (26 alleles; 17.3%); p.l172N (15 alleles; 10%). The rarer mutations were novel one p.R484fsX541 (6 alleles; 4%); p.Q318X (4 alleles; 2.7%) and p.R426C (3 alleles; 2%). The majority of patients (61 cases; 75.3%) were homozygotes. Four cases were compound heterozygous. Thirteen patients had only a heterozygous mutation detected. Genotype accurately predicted phenotype in 93.8 and 100% of patients with salt-wasting and simple virilizing, respectively.

The spectrum of mutations of the *CYP21A2* gene in Vietnamese patients is comparable to the some reported in other Asian population. Large deletion accounts for nearly one-third of the genetic defects. Therefore, laboratory should include methods for detecting point mutations as well as large deletions. Genotype-Phenotype correlation was high in the studied patients.

